# Etiology and Outcome of Candidemia in Neonates and Children in Europe: An 11-year Multinational Retrospective Study

**DOI:** 10.1097/INF.0000000000002530

**Published:** 2019-11-08

**Authors:** Adilia Warris, Zoi-Dorothea Pana, Andrea Oletto, Rebecca Lundin, Elio Castagnola, Thomas Lehrnbecher, Andreas H. Groll, Emmanuel Roilides

**Affiliations:** From the * MRC Centre for Medical Mycology, Institute of Medical Sciences, University of Aberdeen, Aberdeen, United Kingdom; † Infectious Diseases Unit, 3rd Department of Pediatrics, Faculty of Medicine, Aristotle University 96 School of Health Sciences, Thessaloniki, Greece; ‡ Fondazione PENTA Onlus, Padua, Italy; § Infectious Diseases Unit, IRCCS Istituto Giannina Gaslini Children’s Hospital, Genoa, Italy; ¶ Division of Pediatric Hematology and Oncology, Hospital for Children and Adolescents, Johann Wolfgang Goethe-University, Frankfurt, Germany; ‖ Infectious Disease Research Program, Center for Bone Marrow Transplantation and Department of Pediatric Hematology and Oncology, University Children’s Hospital Münster, Münster, Germany.; ** See Appendix.

**Keywords:** candidemia, Candida spp, neonates, infants, children

## Abstract

**Background:**

Data on Candida bloodstream infections in pediatric patients in Europe are limited. We performed a retrospective multicenter European study of the epidemiology and outcome of neonatal and pediatric candidemia.

**Material and Methods:**

All first positive blood cultures from patients ≤ 18 years of age with candidemia were registered. Patients’ demographic and clinical characteristics and causative Candida species were collected and analyzed. Regression analysis was used to identify factors independently associated with mortality.

**Results:**

One thousand three hundred ninety-five episodes of candidemia (57.8% male) were reported from 23 hospitals in 10 European countries. Of the 1395 episodes, 36.4% occurred in neonates (≤ 44 weeks postmenstrual age), 13.8% in infants (> 44 weeks postmenstrual age to 1 year) and 49.8% in children and adolescents. *Candida albicans* (52.5%) and *Candida parapsilosis* (28%) were the predominant species. A higher proportion of candidemia caused by *C. albicans* was observed among neonatal patients (60.2%) with highest rates of *C. parapsilosis* seen among infants (42%). Children admitted to hematology-oncology wards presented the highest rates of non-albicans Candida species. Candidemia because of *C. albicans* was more frequent than non-albicans Candida in Northern versus Southern Europe (odds ratio, 2.3; 95% confidence interval, 1.8–2.9; *P* < 0.001). The all-cause mortality at 30 days was 14.4%. All-cause mortality was higher among patients admitted to the neonatal or pediatric intensive care units than other wards. Over time, no significant changes in species distribution were observed.

**Conclusions:**

This first multicenter European study shows unique characteristics of the epidemiology of pediatric candidemia. The insights obtained from this study will be useful to guide clinical management and antifungal stewardship.

Bloodstream infections caused by Candida spp. remain a worldwide cause of substantial in-hospital morbidity and mortality and are associated with an increased financial burden.^1–5^ A propensity analysis conducted in the US revealed that candidemia is associated with a mean increase in hospitalization of 21 days, a 10% increase in mortality and almost 92,000 US dollars excess per case in pediatric inpatient hospital costs.^1^ Data have additionally shown that the epidemiology of candidemia differs significantly among pediatric and adult populations, especially in terms of Candida species distribution, underlying conditions, risk factors and outcomes.^5–12^ Most large prospectively designed epidemiology studies have excluded pediatric patients. Therefore, detailed knowledge of the epidemiology of Candida species among pediatric groups is limited. Apart from a few single-center^10,13–16^ or single country^11,17–24^ epidemiologic studies, only one large international pediatric multi-institutional study has been conducted.^6^

European multicenter pediatric data have not been collected. The European Pediatric Mycology Network (http://www.penta-id.org/antimicrobials/epmyn/) aims to address this research gap and to increase knowledge on the epidemiology of invasive fungal disease in neonates and children across Europe. The EUROCANDY study is an initiative of the European Pediatric Mycology Network in partnership with the Penta network (http://www.penta-id.org) with the main objective to characterize the current fungal and clinical epidemiology of candidemia among hospitalized neonates, children and adolescents across Europe during an extended time.

## MATERIAL AND METHODS

### Study Design

We conducted a retrospective multicenter study of pediatric candidemia (≤ 18 years of age) diagnosed between January 2005 and December 2015. European centers were approached based on their previous participation in Penta clinical studies, hospital characteristics and interest in participating. Individual ethics approval was obtained at each site if required by institutional and/or national regulations.

A secure web-based data collection tool was developed for standardized collection of the data using the Research Electronic Data Capture (REDCap) platform (http://www.project-redcap.org), with data stored in a secure server located at Penta Foundation offices in Padua, Italy. Investigators entered data online or on printed data entry forms for off-line data collection initially, followed by entry into the web-based system. All data were anonymized, with each center assigned a unique Penta center identification number and each isolate submitted by each center assigned a unique, consecutive EUROCANDY Isolate ID number within the EUROCANDY individual candidemia isolate database. These codes were used to link hospital and isolate data. Centers with < 10 candidemia entries in the REDCap database were excluded from analysis.

### Data Definition and Data Collection

The secure web-based REDCap database tool enabled the standardized collection of the following data: hospital and individual admission ward characteristics, annual hospital ward admissions, demographics, clinical characteristics, causative Candida species and clinical outcome at day 30. The study period was divided into 2 time periods (2005–2010 and 2011–2015, respectively) to allow for the analysis of trends over time.

The study population was stratified according to age and ward admitted to. Patients were stratified according to age in the following age groups: neonates (postmenstrual age ≤ 44 weeks), infants (postmenstrual age > 44 weeks to 1 year) and children and adolescents (> 1 year to < 18 years of age).

The classification of pediatric wards included general pediatric ward, pediatric intensive care unit (PICU), neonatal intensive care unit (NICU), pediatric hematology-oncology ward (including bone marrow transplant unit), pediatric surgery ward, other ICUs and other designated pediatric wards (eg, pediatric neurology, pediatric gastroenterology).

All first positive blood cultures (BC) from patients ≤ 18 years of age with candidemia were included, while any BC from the same patient positive for the same Candida species within 30 days after the first positive BC was not included. Polyfungal candidemia was defined as two or more Candida species present in the same BC. BCs with a different Candida species independent of the time interval to the first positive BC were included as a new episode. A new BC with the same Candida species after an interval of 30 days was regarded as a separate candidemia episode and included as such in the database. Information with respect to negative follow-up BC was not collected, and no differentiation was made between a relapse or reinfection.

### Statistical Analysis

Demographic, clinical and outcome variables were summarized using frequency, percentage, median and first to third interquartile range (IQR). Categorical variables were compared using the χ^2^ or Fisher exact test and continuous variables by the Mann-Whitney *U* test. A 2-sided *P* < 0.05 was considered significant. Differences between the qualitative variables in 2 or more groups were analyzed by χ^2^ test in univariate analysis. Binary regression analysis was used to identify factors that were significantly associated with mortality. Clinically relevant indices in the univariate analysis (*P* < 0.1) were included in a multivariate regression analysis. A multivariable model was identified using backward stepwise variable selection methods. Statistical analysis was conducted using SPSS (IBM SPSS Statistical package for sciences version 23.0, IBM SPSS Inc., New York).

## RESULTS

### Patient Enrollment

Over the 11-year study period (2005–2015), 1395 pediatric candidemia episodes from 23 participating European centers from 10 countries [Belgium (n = 1), Denmark (n = 2), Germany (n = 3), Greece (n = 2), Italy (n = 4), the Netherlands (n = 2), Norway (n = 1), Serbia (n = 1), Spain (n = 3), United Kingdom (n = 4)] were included. Four centers reported < 10 candidemias and were excluded from analysis. A total of 59.1% (n = 824) were reported from Northern European countries (defined as the United Kingdom, the Netherlands, Belgium, Germany, Denmark and Norway). The median number of candidemia episodes entered per country was 107 (range: 40–401; IQR: 108). There was no significant difference between the mean number of episodes per country and geographic area of the centers [Northern vs. Southern European countries, 137.5 vs. 142.7 (mean) episodes per country (*P* = 0.9)].

The median number of episodes entered per year was 125 (range: 95–166; IQR: 31). Between the 2 study periods (2005–2010 and 2011–2015), there was no significant difference in the median number of candidemias reported (first-period median number 125 vs. second period 121 episodes per year).

### Demographic Characteristics

The demographic characteristics of the total cohort are shown in Table 1. The median age was 38 months (range: 1–216; IQR: 95) with a slight predominance of male sex (57.8%). The distribution of episodes in the age categories was as follows: 507 episodes (36.4%) in neonates, 193 episodes (13.8%) in infants and 695 episodes (49.8%) in children.

**TABLE 1.**
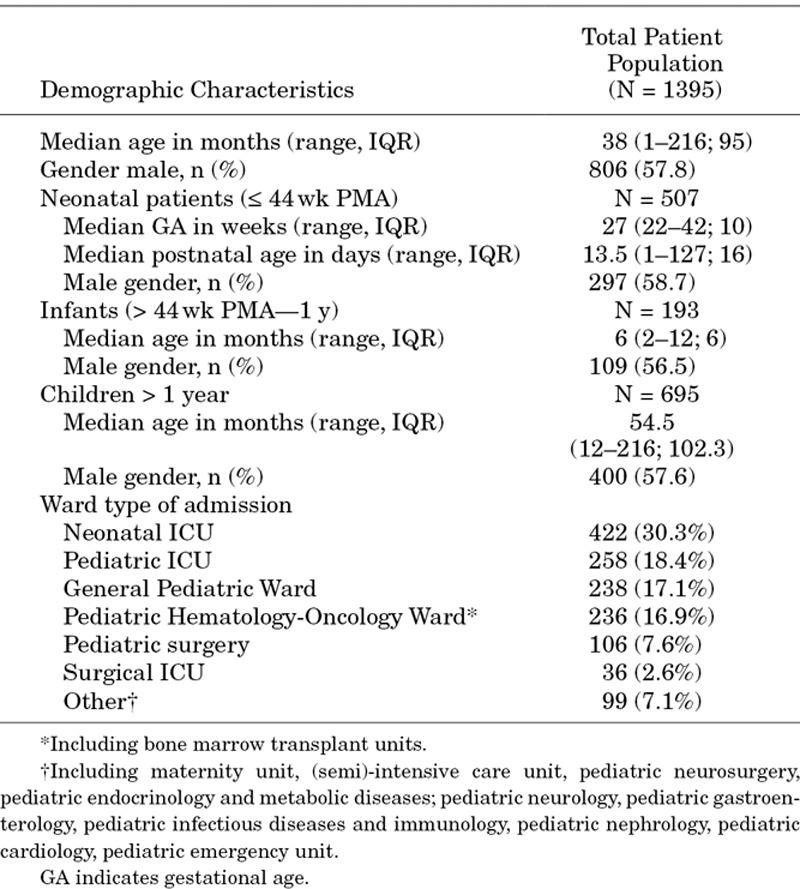
Demographics of Neonatal and Pediatric Patients With Candidemia During the Study Period (2005–2015)

For the neonatal patients, the median gestational age was 27 weeks (range: 22–42 weeks; IQR: 10) with a median postnatal age of 13.5 days (range: 1–127; IQR: 16). For the infant group, the median age was 6 months (range: 2–12; IQR: 6), while for the pediatric group, the median age was 4.5 years (range: 1.2–18; IQR: 5.7).

The majority of the patients were admitted to either the NICU, PICU, general pediatric or hematology-oncology unit, with almost one-third being admitted to the NICU (Table 1).

### Candida Species Distribution

*Candida albicans* was isolated in half of the candidemia episodes (52.6%; n = 734), followed by *C. parapsilosis* (28.1%; n = 392). *Candida tropicalis*, *Candida glabrata* and *Candida krusei* were isolated in 4.4%, 3.5% and 2.2% of the episodes, respectively. Nine episodes of candidemia were caused by more than one Candida species (0.7%). *C. albicans* was shown to be the leading species causing candidemia independently of the ward type, with *C. parapsilosis* being second to *C. albicans* (Table 2). A higher proportion of candidemia caused by non-albicans Candida species was reported in patients admitted to pediatric hematology-oncology wards (60.2%) and other designated pediatrics wards (59.6%) (Table 2).

**TABLE 2.**
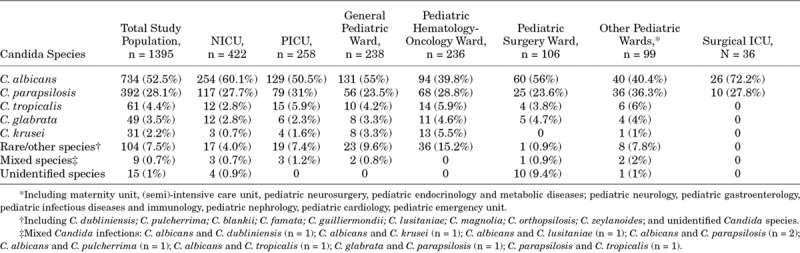
Distribution of *Candida* Species Causing Neonatal and Pediatric Candidemia in Specific Wards Where the Patients Were Diagnosed With Candidemia

A variation in the distribution of C*. albicans* was observed according to the age of the patients, with higher proportion among neonatal patients (60.2%) versus the other 2 age groups (48.7% and 48.2%). Highest rates of *C. parapsilosis* were present among infants (42%), with clearly less candidemias caused by *C. parapsilosis* in neonates (26%) and children (25.8%) (*P* < 0.05; 95% confidence interval, 0.23–0.36). Focusing on individual patient ward populations, the distribution of *C. albicans* versus non-albicans Candida candidemia in the NICU setting was different between the 2 periods (Table 3). A significant difference in the number of *C. parapsilosis* infections was observed between Northern and Southern European countries (16.4% vs. 35.7%; *P* < 0.001). An increase in the proportion of *C. albicans* versus *C. parapsilosis* candidemia episodes in the NICUs was observed over time [55.5% (2005–2010) to 65.5% (2011–2015); *P* = 0.03].

**TABLE 3.**
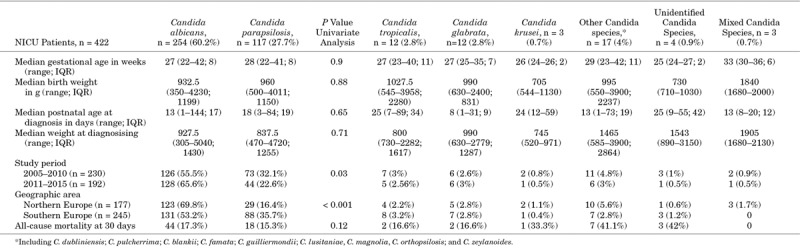
Characteristics and *Candida* Species Distribution Among Patients Admitted to the NICU

The proportion of *C. albicans* versus non-albicans Candida species causing candidemia was different between Northern and Southern European countries with higher proportions of *C. albicans* reported in the Northern countries (58.6% vs. 44%; *P* = 0.001) (Table 4). No differences were observed in the relative number of candidemia episodes caused by *C. albicans* (51.7% vs. 53.7%) and non-albicans Candida (48.3% vs. 46.3%) between the 2 periods (2005–2010 vs. 2011–2015) (Table 4).

**TABLE 4.**
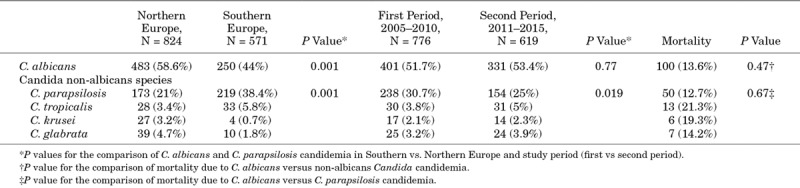
Comparison of C. albicans Versus non-*albicans Candida* spp. Candidemia Distribution From Univariate Analysis

### Outcome

All-cause mortality at 30 days was 14.4% (n = 201), with non-significant differences between neonates and infants compared with older children (18.2% and 14.5% vs. 11.5%, respectively, *P* > 0.05). Similar mortality rates were observed for *C. albicans* (13.6%) and *C. parapsilosis* (12.7%) candidemia, while higher mortality rates were observed for *C. tropicalis* and *C. krusei* (21.3% and 19.3%, respectively) (Table 4). Higher mortality rates were reported among patients admitted to the PICU (27.8%, *P* < 0.001) and NICU (18.3%, *P* = 0.01). Lower rates were found for patients admitted to general and other pediatric wards (4.7% and 5.8%, respectively) and pediatric surgical wards (1.8%). A higher all-cause mortality rate at 30 days was observed for NICU patients (18.3%; 77/422) versus non-NICU patients 12.7% (124/973). The mortality rates among NICU patients increased, although not significantly, between 2005–2010 and 2011–2015, 35/229 (15.2%) and 41/193 (21.7%), respectively (*P* = 0.127). Higher mortality rates among NICU patients were reported when infected with *C. krusei*, other rare species or polycandidal infections, but patient numbers were low (Table 3). Remarkably, all-cause mortality was higher in the second period of the study, 17.1% versus 12.2% in the first period (*P* = 0.05).

### Multivariate Analysis

In the multivariate analysis, the risk of all-cause mortality at 30 days was higher for patients admitted to the NICU, PICU and pediatric hematology-oncology units (Table 5).

**TABLE 5.**
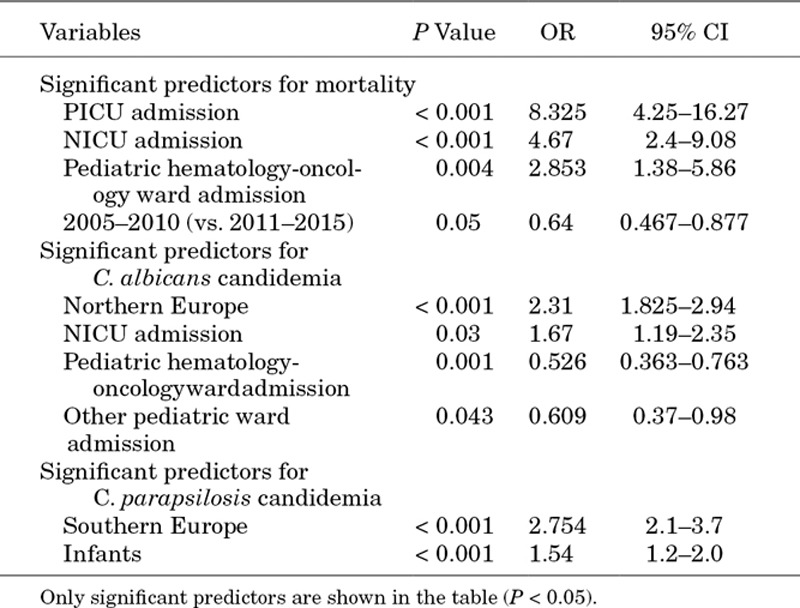
Multivariate Predictors of Mortality and Species Distribution for the EUROCANDY Cohort

When adjusted for confounding factors as age and type of ward admitted to, mortality was lower in the first period (2005–2010) compared with the second period (2011–2015) (*P* = 0.05), the odds of isolating *C. albicans* being the cause of candidemia was 2.3 times more likely in Northern Europe (*P* < 0.001), and the odds of isolating *C. parapsilosis* was 2.7 times higher in Southern Europe (*P* < 0.001). As this finding is driven by the significantly different distribution between those 2 species observed among patients admitted to the NICU, we analyzed the use of neonatal antifungal prophylaxis. The use of neonatal antifungal prophylaxis overall (median number of years/center) did not differ between the 2 regions, but some remarkable differences were noted. Either nystatin or fluconazole (both in 50%) was prescribed in the northern countries, while in southern countries only fluconazole was prescribed. The median numbers of years in which fluconazole prophylaxis was used over the study period was 1.9 y/center (range: 0–11) and 3.9 y/center (range: 0–9 y) for Northern and Southern Europe countries, respectively. The number of centers prescribing routinely neonatal fluconazole prophylaxis increased from 1 center in 2005 to 6 centers in 2015.

## DISCUSSION

To the best of our knowledge, this is the largest European multicenter epidemiologic study on candidemia in neonatal and pediatric patients, including 23 sites from 10 different European countries. Among 1395 candidemia episodes included in the EUROCANDY study, *C. albicans* prevailed followed by *C. parapsilosis*. The distribution of *C. albicans* versus non-albicans Candida species did not differ significantly among the 2 study periods (2005–2010 vs. 2011–2015). Comparing the overall distribution of Candida isolates causing candidemia between Northern and Southern European centers, a significantly higher proportion of *C. albicans* was noted in the Northern countries. Among the different pediatric wards, children admitted to hematology-oncology wards presented the highest rates of non-albicans Candida species. A higher all-cause mortality was observed for NICU patients than for non-NICU patients.

Previously published studies have tried to elucidate the contemporary epidemiology of Candida infections over time among different pediatric groups and different geographic regions [1, 6, 9–16, 20–24]. The largest multinational study so far was performed by the International Pediatric Fungal Network (IPFN) for 2007–2011.^6^ The results of this study, encompassing predominantly centers in the United States (20 United States, 7 European and 3 other international centers), showed that among 221 patients, non-albicans Candida species were the predominant cause of invasive candidiasis (56%).^6^ More specifically, focusing on the causative species identified from invasive candidiasis in the non-neonatal pediatric population (201 isolates), *C. albicans* was isolated in 44% followed by *C. parapsilosis* in 22%.^6^ These results are comparable with respect to the non-neonatal pediatric patients (n = 973) in our study, where *C. albicans* was isolated in 49% and *C. parapsilosis* in 28% of the candidemia episodes. Comparison of the etiology of neonatal candidemia between the 2 studies is challenging as only 25 neonates were included in the IPFN study. A more recently published surveillance study in the United States (period 2009–2015) revealed that among 307 pediatric candidemia cases, *C. albicans* prevailed in neonates (68%) and was the single most common species found in infants and older children,^8^ which is very similar to our findings. Single country studies have shown country-specific differences with Slovakia, Turkey, Norway, Denmark and the United Kingdom^19,21,23,24^ showing lower incidences of *C. parapsilosis* (10%–31%) in neonates and children, compared with Spain, Australia and Latin America (26%–63%).^11,17,20^

We observed a higher incidence of *C. parapsilosis* (42%) among infants, while for neonates only 28% of candidemias were caused by this species. The predominance of *C. parapsilosis* in this specific age group has been previously reported in Spain, with 63.4% of candidemia in infants being caused by *C. parapsilosis*.^17^ A prospective pediatric study (302 patients) from Latin America showed a slightly higher incidence of *C. parapsilosis* in neonatal patients compared with non-neonatal pediatric patients (36% vs. 26%).^11^ A prospective study performed in Australia showed high incidences for *C. parapsilosis* candidemia in both the neonatal and non-neonatal pediatric patients, 42% and 38%, respectively.^20^ A pediatric surveillance study performed in England and Wales, age-specific Candida species distribution causing bloodstream infections was reported.^21^ In this large study, encompassing 706 neonates and infants < 1 year of age, *C. parapsilosis* caused 23% of the neonatal candidemias and 16% of the candidemias in infants.^21^ These differences among the neonatal and pediatric groups might reflect different clinical practices and interventions among different pediatric age groups. In addition, we may have been able to observe the higher prevalence of *C. parapsilosis* in infants due to the high number of pediatric patients included, enabling us to differentiate in greater detail age-specific differences.

Our study showed that the rate of *C. albicans* as cause of candidemia was significantly higher in Northern European centers, while the relative frequencies of *C. parapsilosis* and *C. tropicalis* candidemia were significantly higher in Southern ones. This results from the significant difference in species distribution of those 2 species among NICU patients between the 2 geographic areas. Neonatal fluconazole prophylaxis was significantly higher in Southern European centers compared with Northern ones, but whether this is driving the observed differences remains unclear. Fluconazole prophylaxis in adult patients has been associated with the emergence of *C. glabrata* infections, but no associations with *C. parapsilosis* infections have been reported.^25^ A single country multicenter cohort study among 423 extreme low birth weight neonates showed fluconazole prophylaxis increased the incidence of infections caused by fluconazole-resistant *C. parapsilosis* (0% vs. 41.7%).^26^ A study to analyze the susceptibility profiles of the *Candida* isolates reported in this study is currently being undertaken.

Few previously published candidemia surveillance studies have shown that irrespective of age, *C. albicans* remains the most important causative agent of candidemia among Northern European countries.^18^ A higher incidence of *C. parapsilosis* candidemia in South-Europe, Mediterranean countries and Latin America has been observed in several population- and hospital-based studies (reviewed in ^27^). These observations are of high clinical importance and need to be further studied in association with possible differences in the health care system practices across Europe, as well as the local/national antimicrobial/antifungal stewardship programs.

The all-cause mortality rate at 30 days in our study was 14.4%, which is comparable to the pediatric studies performed in the United States and the United Kingdom^8,21^ and slightly lower compared with the rates reported in the IPFN multicenter study.^6^ In contrast, mortality rates for neonatal and pediatric candidemia were much higher in the study performed in Latin America, including 302 patients.^18^ An explanation for this may lie in the fact that in this study, 12.3% of the patients did not receive any antifungal treatment.^18^ In line with our results, 3 previous studies also showed a higher mortality rate among neonates compared with older infants and children.^6,11,20^ As the majority of the neonates are prematurely born and admitted to a NICU, the overall severity of disease is most likely the explanation for this. The same holds true for children admitted to the PICU and developing candidemia with a less favorable outcome compared with children admitted to non-ICU units,^16^ although not every study has shown this trend.^22^ The higher mortality rate among neonates in the second time period of our study is of concern. Part of the explanation may be sought in the progress in advanced critical care medicine allowing extremely premature infants to survive. The risk for non-survival in our cohort was almost 2 times higher for candidemia due to rare Candida species compared with *C. albicans* candidemia. This observation needs to be further evaluated in association with other potentially relevant factors such as initial treatment prescribed and the differences in virulence and/or antifungal susceptibility profiles of *C. albicans* versus more rare *Candida* species.

Certain limitations of our EUROCANDY study need to be addressed. First, a selection bias is possible since the retrospective design of the study cannot ensure the complete capture of all candidemia episodes at each participating center. We aimed to eliminate the risk of having a “non-representative European sample” of candidemia episodes over the defined time period by excluding centers with < 10 candidemia entries or centers located outside Europe. In addition, the study population consisted of a heterogenic group of pediatric patients and the stratification approach was based on age and ward type on admission, both indicating indirectly the underlying conditions rendering them at risk to develop candidemia. Specific neonatal risk factors as gestational age and birth weight were collected, but other specific known risk factors were not obtained as this was technically not feasible. Finally, candidemia-attributable mortality data was not available. However, the large number of episodes and the broad representation of European centers and length of time allowed us to draw important conclusions. Second, each center participating in the EUROCANDY study contributed differently to the results of the study in term of number of inclusions. Although we aimed to collect the total admissions rates of the specific pediatric wards over the study period, relatively few centers were able to provide these denominators, which precluded the calculation of incidence rates per center. Future studies are needed to capture the incidence rates of pediatric candidemia, the antifungal susceptibilities of the Candida isolates, the antifungal prophylaxis regimens used and their influence on local/national fungal epidemiology, as well as the existing pediatric antifungal stewardship programs across Europe.

The presented results have provided us with an extended insight in the clinical and fungal epidemiology of candidemia in neonates and children among geographically different areas in Europe over an extended time period. The observed differences between Northern and Southern European countries and trends between 2005 and 2010 versus 2010 and 2015, point towards potential differences in infection prevention measures, management of underlying conditions, antifungal use, antimicrobial and antifungal stewardship policies, and need further exploration. The ward-specific fungal epidemiology needs to be addressed in clinical management guidelines. Overall, the results of the EUROCANDY study will allow development of local, national and European clinical management guidelines and to target pediatric antifungal stewardship programs.

## ACKNOWLEDGMENTS

We are grateful for the support provided by the Penta Foundation. A.W. is supported by the Wellcome Trust Strategic Award (grant 097377) and the MRC Centre for Medical Mycology (grant MR/N006364/1) at the University of Aberdeen. Z.-D.P. was supported by a 2017–2019 European Society for Pediatric Infectious Disease (ESPID) Fellowship Award.

## APPENDIX

**EUROCANDY study group:

Cecilie T. Andersen, Oslo University Hospital, Oslo, Norway.

Maiken C. Arendrup, Unit of Mycology, Statens Serum Institut, and Dept of Clinical Microbiology, Rigshospitalet, and Dept of Clinical Medicine, University of Copenhagen, Copenhagen, Denmark.

Valentina Arsic Arsenijevic, Faculty of Medicine University of Belgrade Institute of Microbiology and Immunology Medical Mycology Reference Laboratory, Belgrade, Serbia.

Sonia Bianchini, Fondazione IRCCS, Ospedale Maggiore Policlinico, Milan, Italy.

Ulrich von Both, Children’s Hospital, University Hospital, Ludwig Maximilians University (LMU) Munich, Germany; German Centre for Infection Research (DZIF), partner site Munich.

Martin Chmelnik, Hospital for Children and Adolescents, Johann Wolfgang Goethe-University Frankfurt, Germany.

Tiziana Controzzi, Azienda Ospedaliero-Universitaria Pisana, Pisa, Italy.

Marieke Emonts, Great North Children’s Hospital- Newcastle upon Tyne Hospitals NHS Foundation Trust, Pediatric Immunology- Infectious Diseases and Allergy, and Newcastle University, Institute of Cellular Medicine, Newcastle upon Tyne, United Kingdom.

Susanna Esposito, Pediatric Clinic, Department of Surgical and Biomedical Sciences, University of Perugia, Perugia, Italy and Pietro Barilla Children’s Hospital, Department of Medicine and Surgery, University of Parma, Parma, Italy.

Laura Ferreras-Antolin, St Georges University Hospitals, NHS Foundation Trust, London, United Kingdom.

Stefanie Henriet, Amalia Children’s Hospital, Radboud University Medical Center, Nijmegen, the Netherlands.

Elias Iosifidis, Hippokration Hospital, Thessaloniki, Greece.

Adam Irwin, Great Ormond Street Hospital London, United Kingdom, University of Queensland Centre for Clinical Research, Australia.

John Kopsidas, Centre for Clinical Epidemiology and Outcomes Research (CLEO), Athens, Greece.

Katrien Lagrou, Department of Laboratory Medicine, University Hospitals Leuven, Belgium.

Hermione Lyall, St Mary’s Hospital London, United Kingdom.

Angela Manzanares Casteleiro, Department of Pediatrics, Hospital 12 de Octubre, Fundación para la Investigación Biomédica del Hospital Universitario 12 de Octubre, Madrid, Spain.

Alessio Mesini, Infectious Diseases Unit, IRCCS Istituto Giannina Gaslini Children’s Hospital, Genoa, Italy.

Peter Olbrich, Hospital Universitario Virgen del Rocío, Seville, Spain.

Stephane Paulus, Institute of Infection and Global Health, University of Liverpool, United Kingdom.

Karen Rokkedal Lausch, Aarhus University Hospital, Aarhus, Denmark.

Pere Soler-Palacin, Hospital Universitari Vall d’Hebron, Barcelona, Catalonia, Spain.

Nikos Spyridis, Aglaia Kyriakou Children’s Hospital Athens, Greece.

Volker Strenger, Department for Pediatrics and Adolescent Medicine, Medical University Graz, Graz, Austria.

Martha Theodoraki, Neonatal Intensive Care Unit, General Hospital of Nikea and Pireus, Athens, Greece.

Tom Wolfs, Wilhelmina Children’s Hospital, Utrecht, the Netherlands.
